# Exenatide Improves Bone Quality in a Murine Model of Genetically Inherited Type 2 Diabetes Mellitus

**DOI:** 10.3389/fendo.2017.00327

**Published:** 2017-11-20

**Authors:** Marie Pereira, Stephanie Gohin, Jean-Paul Roux, Amy Fisher, Mark E. Cleasby, Guillaume Mabilleau, Chantal Chenu

**Affiliations:** ^1^Department of Comparative Biomedical Sciences, Royal Veterinary College, London, United Kingdom; ^2^INSERM UMR1033, Université de Lyon, Lyon, France; ^3^Transpharmation, London, United Kingdom; ^4^GEROM-LHEA UPRES EA 4658, Institut de Biologie en Santé, Université d’Angers, Angers, France

**Keywords:** exenatide, type 2 diabetes, db/db mice, skeleton, bone quality, blood flow

## Abstract

Type 2 diabetes mellitus (T2DM) is associated with skeletal complications, including an increased risk of fractures. Reduced blood supply and bone strength may contribute to this skeletal fragility. We hypothesized that long-term administration of Exenatide, a glucagon-like peptide-1 receptor agonist, would improve bone architecture and strength of T2DM mice by increasing blood flow to bone, thereby stimulating bone formation. In this study, we used a model of obesity and severe T2DM, the leptin receptor-deficient db/db mouse to assess alterations in bone quality and hindlimb blood flow and to examine the beneficial effects of 4 weeks administration of Exenatide. As expected, diabetic mice showed marked alterations in bone structure, remodeling and strength, and basal vascular tone compared with lean mice. Exenatide treatment improved trabecular bone mass and architecture by increasing bone formation rate, but only in diabetic mice. Although there was no effect on hindlimb perfusion at the end of this treatment, Exenatide administration acutely increased tibial blood flow. While Exenatide treatment did not restore the impaired bone strength, intrinsic properties of the matrix, such as collagen maturity, were improved. The effects of Exenatide on *in vitro* bone formation were further investigated in primary osteoblasts cultured under high-glucose conditions, showing that Exenatide reversed the impairment in bone formation induced by glucose. In conclusion, Exenatide improves trabecular bone mass by increasing bone formation and could protect against the development of skeletal complications associated with T2DM.

## Introduction

Bone fragility is an important complication in patients with type 2 diabetes mellitus (T2DM), despite a normal or high-bone mineral density (BMD) (1), implying alterations of bone quality (2). The cellular and molecular mechanisms leading to the reduced bone strength and quality in T2DM patients are poorly characterized; but accumulation of advanced glycation end products, as a result of hyperglycemia and oxidative stress, changes in collagen cross-linking and suppression of bone turnover are thought to be significant contributors in the etiology of diabetic fractures (3, 4).

Glycemic control has been evidenced as a major intervention to reduce fracture in T2DM (5), suggesting that early lifestyle intervention and administration of anti-diabetic medications to reduce hyperglycemia are required. However, the choice of anti-diabetic medications is crucial as some anti-diabetic therapies can themselves result in increase in fracture risk by augmenting the risk of hypoglycemia and falls or by altering bone turnover and bone quality.

Incretin hormones, such as glucagon-like peptide-1 (GLP-1) and glucose-dependent insulinotropic peptide (GIP), are peptides secreted in the gastrointestinal tract that stimulate insulin secretion in response to the ingestion of nutrients in a glucose-dependent manner and as such avoidance of hypoglycemia events (6). In opposition to GIP, GLP-1 resistance does not seem to occur in T2DM and as a result, GLP-1 receptor agonists (GLP-1RAs), including Exenatide, have been developed and marketed for the treatment of T2DM (6). A growing body of evidence suggest positive effects of GLP-1 or GLP-1RAs on bone physiology as demonstrated by rapid changes in gene expression or bone mass in several rodent models of ovariectomy-induced osteoporosis, genetically inherited or high-fat diet-induced T2DM (7–11). However, the possible effects of GLP-1RAs on bone quality that is compromised in T2DM, is still lacking. Based on evidences that Glp1r KO mice and GLP-1RA-treated osteoporotic mice, exhibit modifications of bone quality (12–14), we hypothesized that GLP-1RAs could improve bone quality in T2DM.

Furthermore, bone perfusion strongly correlates with metabolic activity and bone formation (15). This is dependent upon the number and size of blood vessels, which are regulated through the processes of angiogenesis and vasomotor function, respectively. Sufficient blood supply is crucial for bone formation, bone turnover, and fracture healing, poor blood supply being a major cause of impaired bone formation and delayed bone healing in the elderly and in osteoporosis (16, 17). T2DM is often associated with impaired vascular function (18) and a high risk of vascular disorders (19). Interestingly, it has been shown that administration of GLP-1RAs to T2DM patients attenuates hypertension, increases renal blood flow, and improves vascular endothelial function (20). Moreover, GLP-1R is expressed in endothelial and vascular smooth muscle cells (21), suggesting that GLP-1RAs may increase blood flow to bone, thereby stimulating bone formation.

The diabetic db/db mouse is a widely used model of severe T2DM because of its shared features with human diabetes (22). These mice possess a mutation in the adipokine leptin receptor gene that causes obesity and subsequent spontaneous development of T2DM-like pathology, including early hyperlipidemia and hyperinsulinemia, followed by persistent hyperglycemia and decreased insulin secretion, as a result of pancreatic β-cell dysfunction (23). db/db mice exhibit skeletal fragility compared with their lean controls (22), in particular impaired trabecular and cortical microarchitectures and reduced bone strength due to poor bone quality (22, 24, 25). Interestingly, an analog of the sister incretin hormone GIP has been shown to significantly improve bone quality and hence bone strength in the db/db mice (25). In the shed of these results, it is clinically important to assess the effects of marketed GLP-1RAs on bone strength and quality in this rodent model.

The aims of the present study were to ascertain whether Exenatide can improve bone quality and strength in diabetic mice by studying bone microarchitectures, tissue material properties, cellular activities, and hindlimb blood flow *in vivo*. We also compared the effects of Exenatide on *in vitro* bone formation by osteoblasts cultured in low- and high-glucose environments.

## Materials and Methods

### Animals and Study Design

Twenty male diabetic (db/db) mice (BKS.Cg-+Lepr^db^/+Lepr^db^/OlaHsd) and 20 male lean mice (BKS.Cg-Dock7m+/+Leprdb/OlaHsd) were purchased from Harlan Laboratories (Shardlow, UK) at 6 weeks of age and acclimatized to their housing (21 ± 1°C; 12 h day/night cycle) for 1 week. They were weighed and blood glucose measured at the end of each week using a glucometer (Accu-chek Aviva, Roche Diagnostics, UK). All mice were diabetic [blood glucose > 12 mmol/L (26)] at 9 weeks of age. Then, both lean and db/db mice were randomly allocated to treatment with saline (saline control and diabetic control) or 10 μg/kg/day Exenatide (Bachem, Switzerland) (*n* = 10/group). Sample size calculations were based on previous work comparing Exenatide’s effect to saline and db/db mice to lean mice on the most relevant parameter for bone mass, the bone volume percentage (BV/TV) (14, 25) to achieve 80% power of detecting the difference with 5% of type 1 error rate.

Treatments were administered by daily subcutaneous injections for 4 weeks. At days 8 and 3 prior to euthanasia, mice were intraperitoneally injected with calcein (20 mg/kg) and alizarin red complexone (30 mg/kg) (Sigma, UK), respectively, to label bone-mineralizing surfaces. After necropsy, tibias and femurs were collected and cleaned of soft tissues. All animal procedures were approved by the institutional Ethics and Welfare committee and were carried out under UK Home Office license to comply with the Animals (Scientific Procedures) Act 1986 (PPL: 70/7859).

### Measurements of Whole Hindlimb Bone Perfusion in db/db Mice

Two days before blood flow measurement, the hair of the right hindlimb of db/db mice was removed with a depilatory cream. Perfusion was measured in the right hindlimb using laser Doppler imaging (LDI) (MoorLDI2 Imager, Moor Instruments Ltd., UK). The animals were placed in the supine position during LDI measurement and the mean perfusion was estimated from the thigh to the foot to cover the tibia artery and vein. The distance between the camera and the hindlimb skin surface was 20 cm.

To analyze the acute effects of Exenatide on hindlimb perfusion, mice were anesthetized using isofluorane, recordings were started 8 min before a subcutaneous injection of Exenatide (baseline) and continued during the following 30 min. LDI measures the distribution of blood flux (combination of velocity and concentration of red blood cells) in arbitrary units for a selected region of interest (19 mm × 27 mm) with a spatial resolution of 100μm/pixel. The estimated maximum depth of detectable vessels (in soft connective tissues) was 2–3 mm (laser wavelength = 830 nm). LDI generates color-coded perfusion maps of larger tissue areas which allow the recording of precisely delimited regions of interest compatible for specific long bone perfusion measurements in mice (27).

### Micro-CT Analysis of Tibiae

Right tibiae were fixed in 4% formaldehyde for 48 h and stored in 70% ethanol at 4°C. To analyze bone microarchitecture, trabecular and cortical compartments were scanned using high-resolution micro-computed tomography (Skyscan-1172/F Bruker, Belgium) operated at 55 kV, 180 µA, 1,710 ms integration time. The isotropic voxel size was fixed at 5 µm, the rotation step at 0.6°, and exposure was performed using a 0.5 mm aluminum filter. After scanning, whole tibiae were reconstructed using NRecon version 1.6.9.8 (Skyscan), trabecular and cortical bone areas were analyzed with CT-Analyser version 1.14.4.1 (Skyscan), and the quantification of bone structure was made using the analysis software Batman v.1.14.4.1. Trabecular parameters were assessed in the proximal metaphysis; 0.5 mm below the growth plate was left unanalyzed and the 2 mm following down was analyzed. These included: BV/TV, trabecular thickness (Tb.Th), trabecular number (Tb.N), structure model index (SMI), trabecular bone pattern factor (Tb.Pf), and trabecular separation (Tb.Sp). Analysis of cortical bone in the midshaft diaphysis was performed using a 0.5 mm long segment at 50% of the total tibia length. Parameters consisted of: tissue area (Tt.Ar), tissue perimeter (Tt.Pm), bone area (Ct.Ar), eccentricity (Ecc), maximum bending rigidity (*I*_max_), and cross-sectional thickness (Ct.Th). The threshold values for the micro-CT analysis of trabecular bone were chosen to be between 60 and 255 and between 100 and 255 for cortical bone. All parameters were measured according to guidelines and nomenclature proposed by the American Society for Bone and Mineral Research (ASBMR) (28).

### Bone Histomorphometry

Following micro-CT, tibiae of db/db and lean mice were dehydrated in acetone for 24 h and embedded in methylmethacrylate (MMA, Sigma, UK) at low temperature. Unstained 8-μm thick longitudinal sections were used for fluorescence microscopy to visualize mineralizing surfaces. The extent of the mineralizing surfaces was expressed as the alizarin red-labeled surfaces per unit bone surface (MS/BS). Mineral apposition rate (MAR) and bone formation rate (BFR) were calculated as previously described (14). Alternatively, sections were stained for tartrate-resistant acid phosphatase (TRAP) (Leucognost^®^ SP; Merck, Germany) and counterstained with Mayer’s hematoxylin solution to visualize osteoclasts on trabecular bone, or subjected to Goldner’s staining to quantify trabecular bone microarchitecture parameters. Parameters were measured in the trabecular bone of the metaphysis, using a 1 mm region of interest below the growth plate. Measurements were performed using image analysis software (Explora Nova, La Rochelle, France) and parameters were reported in accordance with the ASBMR nomenclature (29).

### Mechanical Testing

Whole bone strength was assessed by three-point bending performed on the left femurs of db/db and lean mice. Prior to mechanical testing, the femurs were hydrated in saline for 24 h at room temperature. Measurements were performed with a constant span length of 10 mm on an Instron 5942 (Instron, France). Femurs were positioned horizontally, with the anterior surface facing upward, centered on the support, and the pressing force was applied vertically to the bone midshaft. Each bone was tested with a loading speed of 2 mm/min until failure with a 500 N load cell, as reported previously (13). The load-displacement curve was acquired using Bluehill 3 software (Instron) and ultimate load, ultimate displacement, stiffness, and work to fracture were quantified.

### Evaluation of Bone Mineral Density Distribution (BMDD)

Quantitative backscattered electron imaging (qBEI) was employed to determine the BMDD in the MMA-embedded blocks above, as previously described (30). MMA blocks were carbon-coated and observed with a scanning electron microscope (EVO LS10, Carl Zeiss Ltd., Nanterre, France) equipped with a five-quadrant semi-conductor backscattered electron detector and operated at 20 keV, with a probe current of 250 pA and a working distance of 15 mm. The backscattered signal was calibrated as described (25). Four images per sample of cortical bone, centered 4 mm below the growth plate, were imaged at a 200× nominal magnification, corresponding to a pixel size of 0.5 µm. Analyses were performed to obtain variables from the BMDD: Ca_mean_, representing the average calcium concentration, Ca_peak_, the most frequently occurring calcium concentration, and Ca_width_, the width of the histogram at the half maximum level.

### Fourier-Transform Infrared Microscopy (FTIRM) at Sites of Bone Formation

The same MMA-embedded blocks used for histomorphometry were utilized for FTIRM. Longitudinal tibia sections (4-µm thickness) were sandwiched between BaF_2_ optical windows. Spectral analysis was obtained on a Bruker Vertex 70 spectrometer (Bruker Optics, Germany) interfaced with a Bruker Hyperion 3000 infrared microscope. For FTIRM analysis at site of bone formation, 12 spectra on each bone were acquired between the alizarin red and calcein labeling and analyzed with Opus Software (release 6.5, Bruker). The contribution of the embedding MMA and water vapor were corrected prior to baseline correction. Individual spectra were then subjected to curve fitting after obtaining a second derivative, using a commercially available software package (Grams/AI 8.0, Thermofisher Scientific, France). The parameters (25) were: (a) mineral-to-matrix ratio; (b) mineral maturity; (c) the carbonate-to-phosphate ratio; and (d) the collagen maturity index. All relevant calculations were described in Ref. (31).

### *In Vitro* Bone Formation by Primary Osteoblasts

Primary mouse osteoblastic cells were obtained by sequential enzyme digestion of excised calvarial bones from 2-day-old C57BL/6 mice using a three-step process (32). Cells were cultured in minimum essential media (MEM) for 2–3 days at 37°C in 5% CO_2_ until they reached confluence. They were then cultured in six-well trays in MEM supplemented with 2 mM β-glycerophosphate and 50 µg/mL ascorbic acid, containing either normal (5.5 mM) or high- (22 mM) glucose concentrations, using mannitol (22 mM) as an osmotic control. Exenatide (0, 25, 50, and 100 nM) was added to the culture (one plate/treatment). Bone nodule formation by osteoblasts was measured after 28 days of culture. Experiments were terminated by fixing cell layers in 4% paraformaldehyde for 10 min; mineralized bone nodules were visualized and quantified unstained. Bone nodules were scanned at 800 dpi using a high-resolution flat-bed scanner. Binary images of each individual well were then subjected to automated analysis (Image J), using constant “threshold” and “minimum particle” levels, to determine the number and surface area of mineralized bone nodules, as previously described (32).

### Statistics

Data are presented as mean ± SD except for acute blood flow where they are presented as mean ± SEM to facilitate visibility. Multiple comparisons were performed using two-way analysis of variance and Tukey’s multiple comparison *post hoc* test where appropriate. *P* < 0.05 was considered to be statistically significant.

## Results

### Effect of Exenatide on Body Mass, Obesity Status, and Blood Glucose in db/db Mice

**Figure 1:**
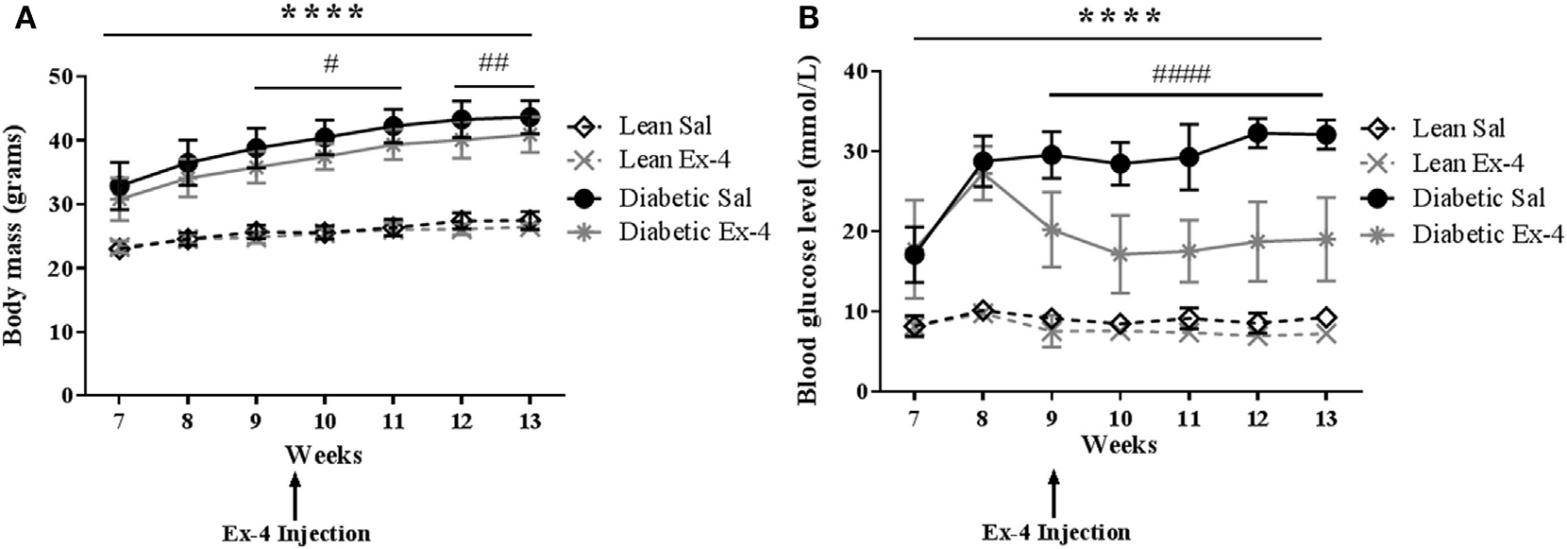
Body mass and blood glucose in db/db and control mice. Body mass and blood glucose levels were measured weekly in db/db and lean mice. Injections of Exenatide started at week 9 and continued daily for 4 weeks. **(A)** Body mass in relation to age in lean, diabetic, and Exenatide-treated mice from 7 to 13 weeks of age. **(B)** Blood glucose levels in lean, diabetic, and Exenatide-treated mice from 7 to 13 weeks of age. Bars represent mean ± SD for *n* = 10 mice/group, ****P < 0.0001 saline-treated diabetic versus saline-treated lean; ^#^*P* < 0.05; ^####^*P* < 0.0001 Exenatide-treated diabetic versus saline-treated diabetic mice.

The development of diabetes in db/db mice was identified by measuring body mass and blood glucose. The body mass of db/db mice was significantly higher than that of lean mice at all time-points, consistent with severe obesity. Administration of Exenatide treatment for 4 weeks slightly reduced this in db/db mice (by 6–7%) compared with saline-treated db/db mice (*P* < 0.01) despite the mice still being obese (Figure 1A). Exenatide had no effect in lean mice (Figure 1A).

Saline-treated db/db mice exhibited dramatically higher blood glucose than saline-treated lean mice at 7–8 weeks [by 2.1-fold (*P* < 0.001)] and at 12–13 weeks [by 3.5-fold (*P* < 0.0001)] (Figure 1B). The mice were all diabetic by 9 weeks of age. These data confirm significant impairment of glycemic control in these mice. Injection of Exenatide into db/db mice decreased the blood glucose levels in the first week of treatment by 31% (*P* < 0.0001) and in the last week by 42% (*P* < 0.0001) compared with saline-treated db/db mice. However, Exenatide had no effect on blood glucose in lean mice.

### Chronic and Acute Effects of Exenatide on Hindlimb Blood Flow in db/db Mice

**Figure 2:**
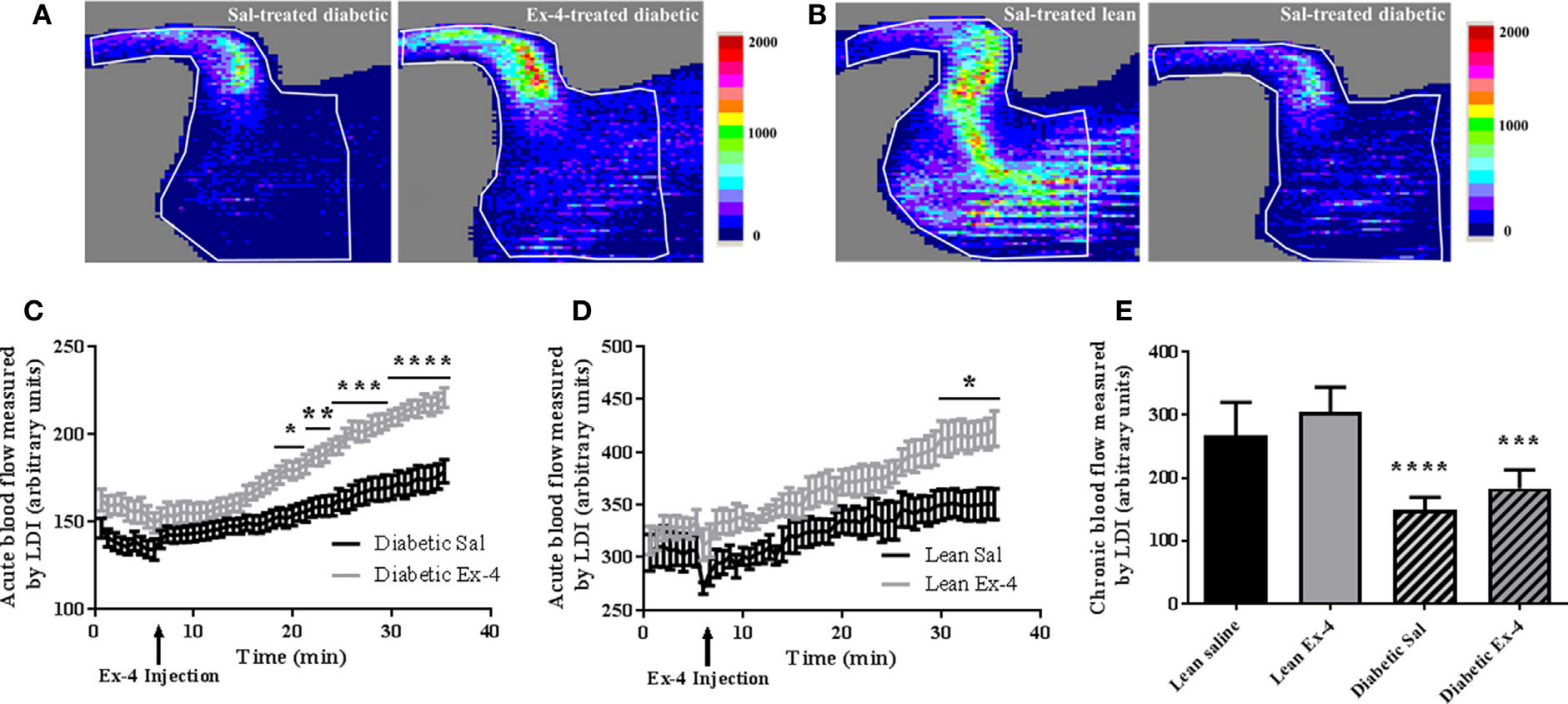
Acute and chronic effects of Exenatide on hindlimb blood flow in db/db mice and control mice. **(A,B)** Representative images of mouse hindlimb perfusion, obtained by laser Doppler imaging (LDI). **(A)** Blood flow imaging of db/db mice with saline injection versus Exenatide. Images were acquired after a 25 min infusion of saline or Exenatide (10 μg/kg/day), **(B)** blood flow imaging of untreated lean mice versus db/db mice. Images were acquired after the 4 weeks of treatment. The colors in the heat maps indicate the maximum (red) and the minimum (blue) levels of perfusion, expressed in arbitrary units, representing the total blood flux (combination of velocity and concentration of red blood cells). **(C,D)** Time course graph of the average hindlimb perfusion monitored by LDI after Exenatide injection in db/db **(C)** and lean **(D)** mice. Exenatide (10 µg/kg) and saline were injected to mice after 8 min of baseline recording. Values are presented as mean ± SEM of *n* = 10 mice/group, *P < 0.05; **P < 0.01; ***P < 0.001; ****P < 0.0001 versus saline. **(E)** Differences in hindlimb blood flow measured by LDI between the groups after 4-week treatment with Exenatide. Bars represent mean ± SD of *n* = 10 mice/group, ****P < 0.0001 versus lean mice.

Acute changes induced by Exenatide in hindlimb perfusion were measured by LDI in all mice. After 15 min injection, Exenatide significantly stimulated blood perfusion in tibiae of db/db mice compared with the saline-treated ones. This vasodilatory effect of Exenatide increased over time for the 30 min following the injection, reaching a maximum 25% (*P* < 0.0001) greater than that in saline-treated db/db mice (Figures 2A,C). This increase in perfusion was also found in Exenatide-treated lean mice (*P* < 0.05) (Figure 2D).

Hindlimb perfusion was 44% lower in db/db mice (*P* < 0.0001) than in lean mice (Figures 2B,E) confirming that diabetic mice have impaired blood flow. The chronic effect of Exenatide on hindlimb perfusion after 4-week treatment was also assessed and our data show no differences in hindlimb perfusion between Exenatide-treated db/db and Exenatide-treated lean mice (Figure 2E). These data indicate that acute but not chronic treatment with Exenatide increases hindlimb perfusion in db/db and lean mice.

### Effect of Exenatide on Trabecular and Cortical Bone Microarchitecture in db/db Mice

We assessed the possible effects of Exenatide on trabecular and cortical bone microarchitecture. As expected, saline-treated db/db mice exhibited lower trabecular bone mass and impaired micro-architectural parameters of connectivity and structure when compared with lean mice (Table 1). This low bone mass in db/db mice was significantly improved by Exenatide treatment, as shown by an increase in BV/TV (+49%, *P* < 0.01) compared with saline-treated db/db mice. This increase was associated with significant modifications of trabecular microarchitecture as evidenced by higher values for Tb.N (+38%, *P* < 0.01) and Tb.Th (+8%, *P* < 0.01) and lower values for microarchitecture, Tb.Pf (−29%; *P* < 0.0001) and SMI (−12%; *P* < 0.01) (Table 1). These beneficial effects of Exenatide in diabetic mice were confirmed by bone histomorphometry performed on 2D sections (Table 1). Tb.Sp was improved by Ex-4 with histomorphometry but not by micro-CT. One reason for this difference can be that measurements made by histomorphometry are performed in 2D, in contrast to micro-CT where there are performed in 3D, and are therefore less accurate. Indeed, in 3D, Tb.Sp is calculated independently of Tb.N and Tb.Th and it has been previously shown that derived Tb.Sp using 2D sections can be different from when measured directly in 3D and may yield in biased results (33).

**Table 1:** Measurements of trabecular and cortical bone architecture in 13-week-old db/db and lean mice treated with either Ex-4 or saline.

Parameters	Lean saline	Lean Ex-4	db/db saline	db/db Ex-4
**Trabecular architecture using micro-CT**				
BV/TV (%)	7.21 ± 1.37	7.83 ± 1.29	3.35 ± 0.51****	5.00 ± 0.78^##^
Tb.Th (μm)	57.4 ± 2.9	58.1 ± 3.3	45.3 ± 2.2****	49.1 ± 1.8^##^
Tb.N (/mm)	1.23 ± 0.24	1.36 ± 0.21	0.74 ± 0.12****	1.02 ± 0.18^##^
Tb.Pf (/mm)	17.00 ± 4.78	15.25 ± 2.72	28.89 ± 2.10****	20.62 ± 2.94^####^
SMI	1.9 ± 0.21	1.78 ± 0.15	2.18 ± 0.09****	1.93 ± 0.13^##^
Tb.Sp (mm)	0.46 ± 0.03	0.45 ± 0.04	0.49 ± 0.03	0.46 ± 0.04
**Trabecular architecture using histomorphometry**				
BV/TV (%)	11.25 ± 3.01	12.86 ± 3.06	4.53 ± 1.30****	7.70 ± 1.21^#^
Tb.Th (μm)	39.5 ± 7.2	39.5 ± 4.3	26.3 ± 3.8****	34.4 ± 4.0^##^
Tb.N (/mm)	3.47 ± 0.39	3.68 ± 0.46	1.68 ± 0.35****	2.31 ± 0.37^##^
Tb.Sp (mm)	0.27 ± 0.05	0.25 ± 0.05	0.59 ± 0.10****	0.44 ± 0.10^##^
**Cortical architecture using micro-CT**				
Tt.Ar (mm^2^)	1.00 ± 0.09	0.97 ± 0.08	0.85 ± 0.08**	0.80 ± 0.09
Tt.Pm (mm)	3.99 ± 0.19	3.93 ± 0.15	3.64 ± 0.18**	3.54 ± 0.18
Ct.Ar (mm^2^)	0.57 ± 0.06	0.55 ± 0.08	0.43 ± 0.06***	0.40 ± 0.07
Ecc	0.55 ± 0.06	0.54 ± 0.07	0.46 ± 0.04*	0.45 ± 0.09
*I*_max_ (mm^4^)	0.14 ± 0.03	0.13 ± 0.03	0.09 ± 0.02***	0.08 ± 0.02
Ct.Th (mm)	0.17 ± 0.01	0.17 ± 0.02	0.14 ± 0.02***	0.13 ± 0.02

*Measurements of trabecular architecture were performed in the proximal tibial metaphysis of db/db and lean mice using micro-CT or histomorphometry. Measurements of cortical architecture were made using micro-CT in the tibial midshaft diaphysis of db/db and lean mice*.

*BV/TV, bone volume percent; Tb.N, trabecular number; Tb.Pf, trabecular pattern factor; Tb.Th, trabecular thickness; Tb.Sp, trabecular separation; SMI, structure model index; Tt.Ar, tissue area; Tt.Pm, tissue perimeter; Ct.Ar, bone area; Ecc, eccentricity; I_max_, maximal moment of inertia; Ct.Th, cross-sectional thickness*.

Mean ± SD of n = 10 mice/group. *P < 0.05, **P < 0.01, ***P < 0.001, ****P < 0.0001 versus lean saline. ^#^P < 0.05, ^##^P < 0.01, ^####^P < 0.0001 versus db/db saline.

Consistent with the trabecular bone changes, cortical bone geometry was also impaired in db/db mice (Table 1). However, our results revealed no differences in any cortical parameter following Exenatide treatment of both lean and db/db mice (Table 1).

### Effect of Exenatide on *In Situ* Bone Turnover in db/db Mice

**Figure 3:**
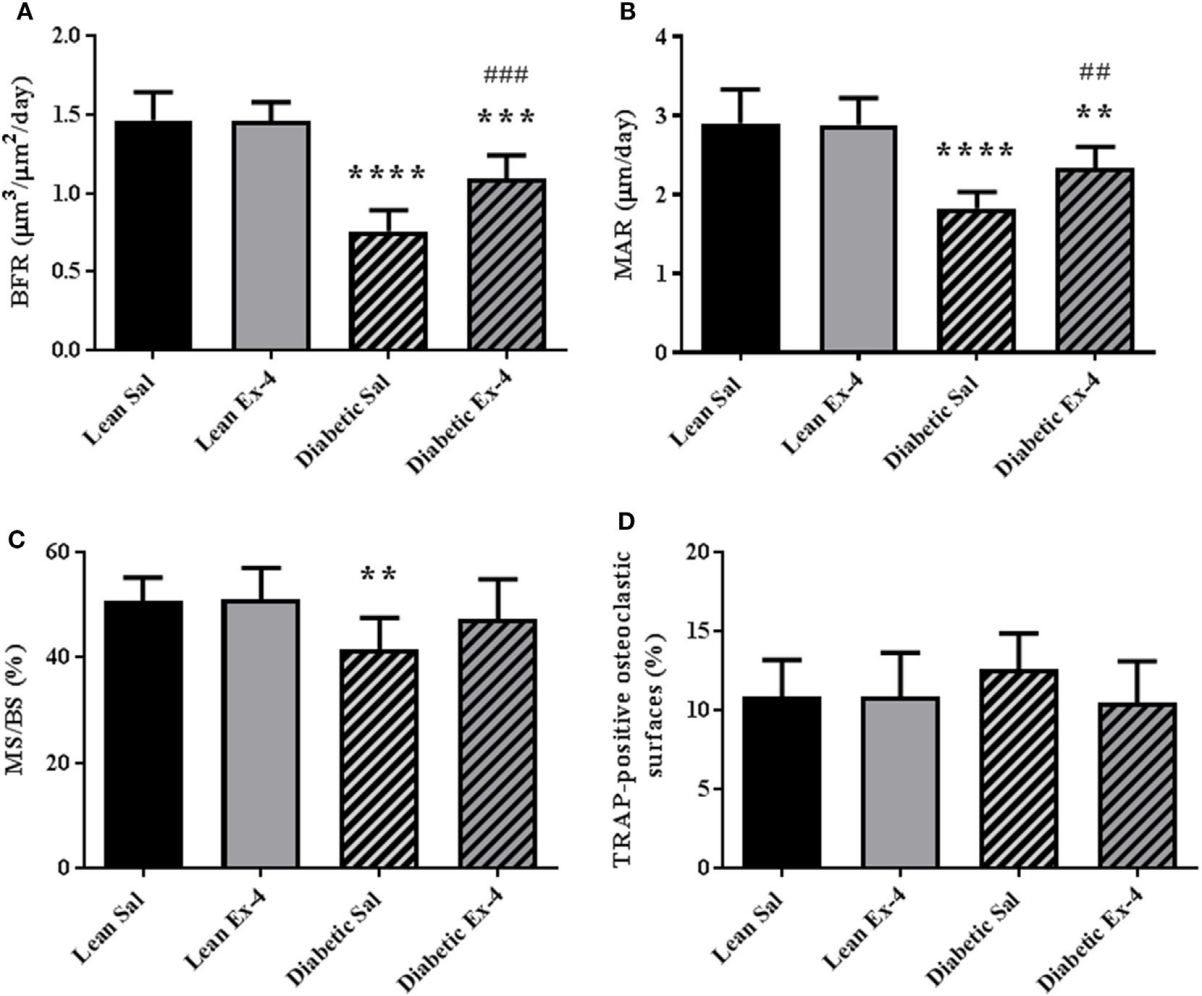
Effect of Exenatide on bone turnover parameters in db/db and control mice. Measurements were performed by bone histomorphometry on the trabecular region of tibial sections from 13-week-old db/db and lean mice treated with either Exenatide (10 μg/kg/day) or saline. **(A)** Bone formation rate (BFR). **(B)** Mineral apposition rate (MAR). **(C)** Mineralizing surfaces MS/BS. **(D)** Tartrate-resistant acid phosphatase (TRAP)-positive osteoclastic surfaces per millimeter of trabecular bone surface. Bars represent mean ± SD of *n* = 10 mice/group, **P < 0.01, ***P < 0.001, ****P < 0.0001 versus lean Sal; ^##^*P* < 0.01, ^###^*P* < 0.001 versus diabetic Sal.

We next examined whether amelioration of trabecular microarchitecture in Exenatide-treated db/db mice was due to a change in osteoblast activity or osteoclast numbers. As expected, BFR/BS was lower in db/db mice than in lean mice. Exenatide treatment increased this parameter by 44.5% in db/db mice (*P* < 0.01) compared with saline-treated ones. This augmentation in BFR/BS was due to higher MAR (+28%; *P* < 0.01) (Figures 3A–C). On the other hand, Exenatide treatment had no effect in lean mice. We did not observe any significant differences in the number of TRAP-positive osteoclastic surfaces among the groups (Figure 3D), suggesting that bone resorption was not affected by diabetes or Exenatide treatment in lean and diabetic animals.

### Effect of Exenatide on *In Vitro* Bone Formation in High-Glucose Conditions

**Figure 4:**
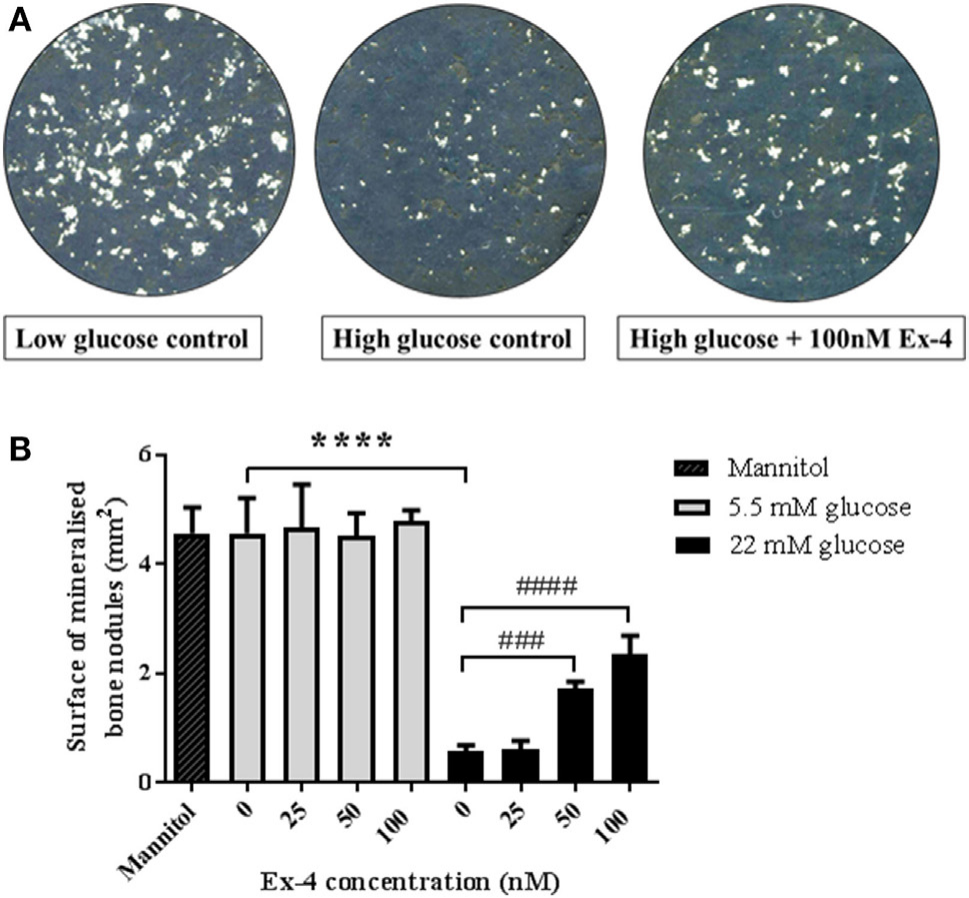
Effects of Exenatide on bone nodule formation *in vitro* under normal and high-glucose conditions. Mouse primary osteoblasts were isolated from calvaria of 2–3 days old pups and cultured for 28 days in six-well plates in osteogenic medium (with addition of ascorbate and β-glycerophosphate) in either normal (5.5 mM) or high- (22 mM) glucose concentration. Exenatide was added to the cells in normal and high-glucose conditions at various concentrations (0, 25, 50, and 100 nM). A control with 22 mM of mannitol was used as an osmotic control to ensure that changes in bone formation is due to glucotoxicity rather than hyperosmolarity. Mineralized nodules representing bone formation were scanned and observed unstained. Quantification of mineralization was performed using Image J. **(A)** Imaging of bone nodules using contrasting color on photoshop to visualize the bone nodules that appear in white. **(B)** Quantification of the area of bone formation by osteoblasts cultured with glucose and Exenatide. Mean ± SD of six wells/group. ****P < 0.0001 versus low glucose condition; ^####^*P* < 0.0001, ^###^*P* < 0.001 versus no Exenatide. Experiments were performed three times in triplicates and graphs are representative of one experiment.

In order to assess whether the above effects were due to direct action of exenatide on bone cells, we investigated the effect of Exenatide on bone formation by primary osteoblasts under high-glucose conditions. Similar to our previous findings (14), we found no effect of Exenatide on bone formation *in vitro* when osteoblasts were cultured in normal glucose concentrations. In contrast, a sevenfold reduction (*P* < 0.0001) in bone nodule formation was observed under high-glucose conditions (Figure 4A). The area of bone nodule formation in the presence of mannitol was similar to that generated in normal glucose conditions, suggesting that the inhibition of bone formation observed in high-glucose medium was not induced by extracellular hyperosmolarity. Exenatide treatment increased the area of bone nodule formation by osteoblasts cultured in high-glucose medium by twofold (*P* < 0.001) and threefold (*P* < 0.0001) with concentrations of 50 and 100 nM, respectively, while it had no effect when osteoblasts were cultured in low-glucose concentrations (Figure 4B).

### Effect of Exenatide on Bone Mechanical Strength in db/db Mice

**Figure 5:**
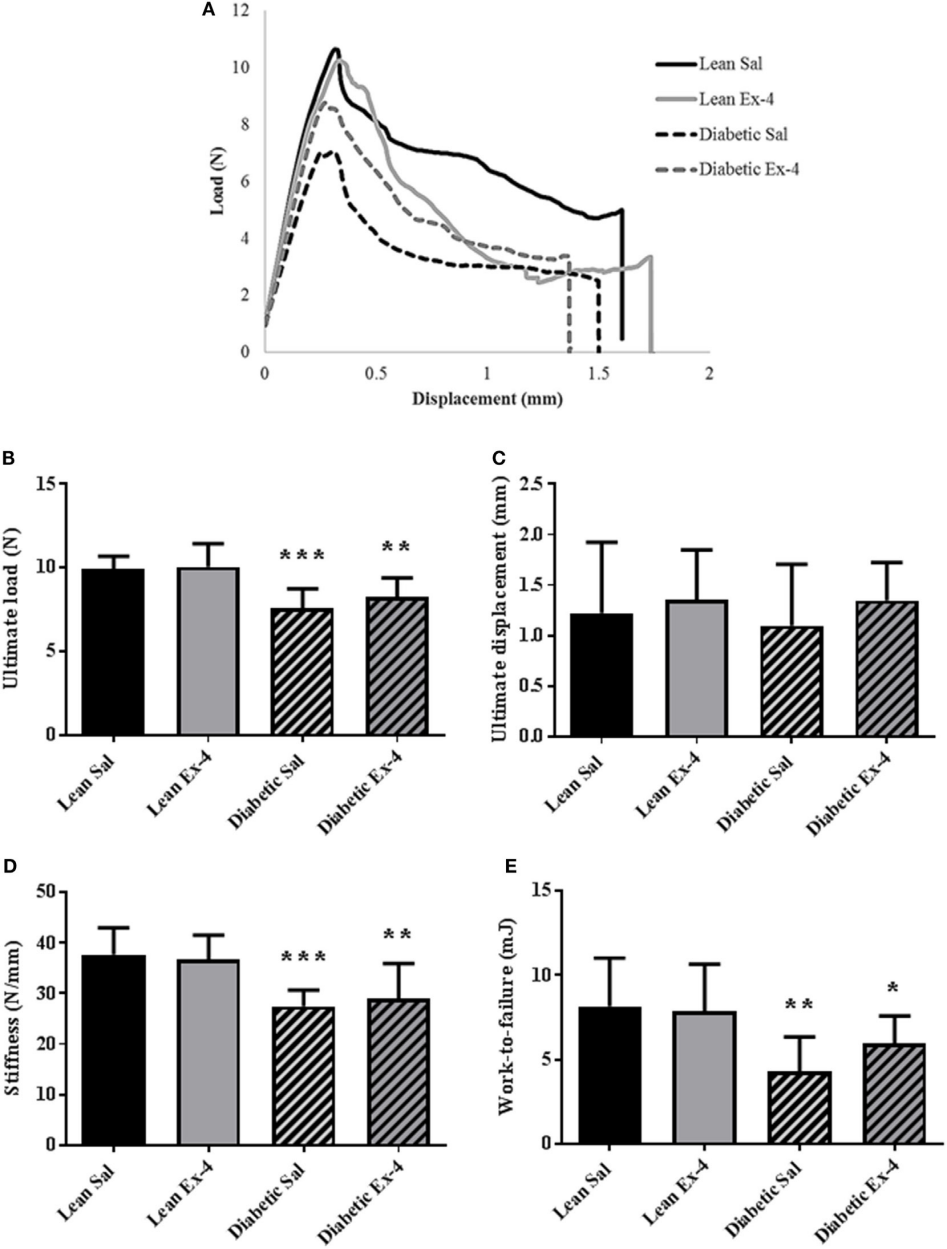
Effect of Exenatide on trabecular bone strength in db/db and control mice. Mechanical parameters were recorded in whole left femur using three-point bending in 13-week-old db/db and lean mice treated with either Exenatide (10 μg/kg/day) or saline. **(A)** Examples of load-displacement curves. **(B)** Ultimate load. **(C)** Ultimate displacement. **(D)** Stiffness. **(E)** Work-to-failure. Bars represent mean ± SD of *n* = 10 mice/group, *P < 0.05, **P < 0.01, ***P < 0.001 versus lean Sal.

Figure 5A represents an example of load/deformation curves that were recorded in the four groups. As expected, db/db mice had compromised bone strength evidenced by reductions in ultimate load, stiffness, and work-to-failure. Treatment of db/db mice with Exenatide led to a slight but not significant increase in ultimate load, stiffness, and work-to-failure (Figure 5).

### Effect of Exenatide on BMDD in db/db Mice

**Figure 6:**
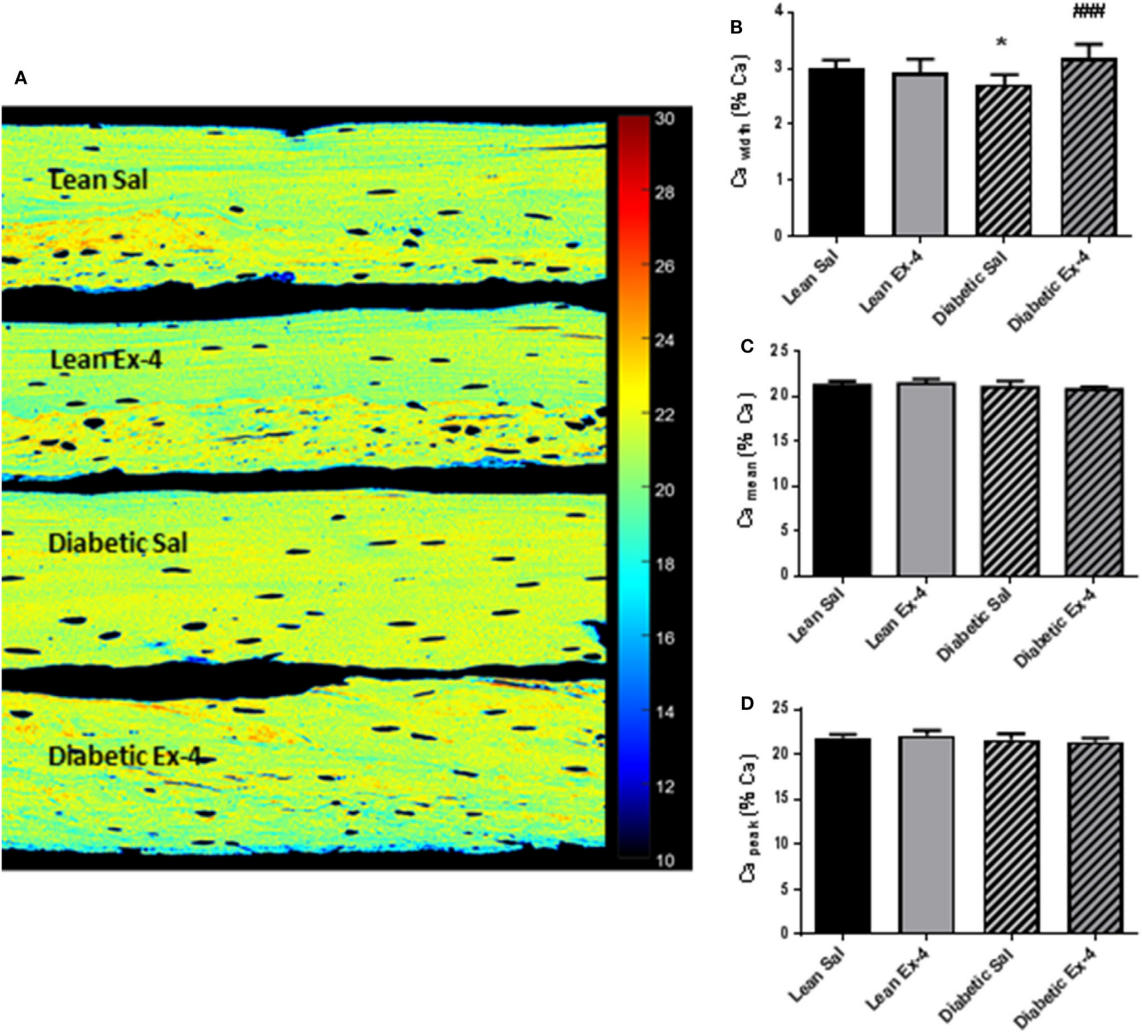
Effect of Exenatide on bone mineral density distribution (BMDD) in db/db and control mice. Quantitative backscattered electron imaging was employed to determine the BMDD in tibial methylmethacrylate-embedded blocks from 13-week-old db/db and lean mice treated with either Exenatide (10 μg/kg/day) or saline. **(A)** Representative calcium (Ca) map. **(B)** Characterization of BMDD by Ca width. **(C)** Characterization of BMDD by Ca mean. **(D)** Characterization of BMDD by Ca peak. Bars represent mean ± SD of *n* = 10 mice/group, *P < 0.05 versus lean Sal; ^###^*P* < 0.001 versus diabetic Sal.

We aimed to look at modifications of the BMDD to see any possible alteration in the calcium content of cortical bone (Figure 6A). Ca_width_, that represents heterogeneity in mineral content, was 9.5% (*P* < 0.05) lower in db/db mice compared with lean mice (Figure 6B). Four weeks’ treatment with Exenatide increased the Ca_width_ by 17% (*P* < 0.001) in db/db mice compared with saline-treated ones (Figure 6B), but had no effect on lean mice. Ca_mean_ and Ca_peak_ were not modified by diabetes or Exenatide treatment (Figures 6C,D), indicating that the average calcium concentration was not affected by either.

### Effect of Exenatide on Tissue Material Properties at Bone Formation Sites in db/db Mice

**Figure 7:**
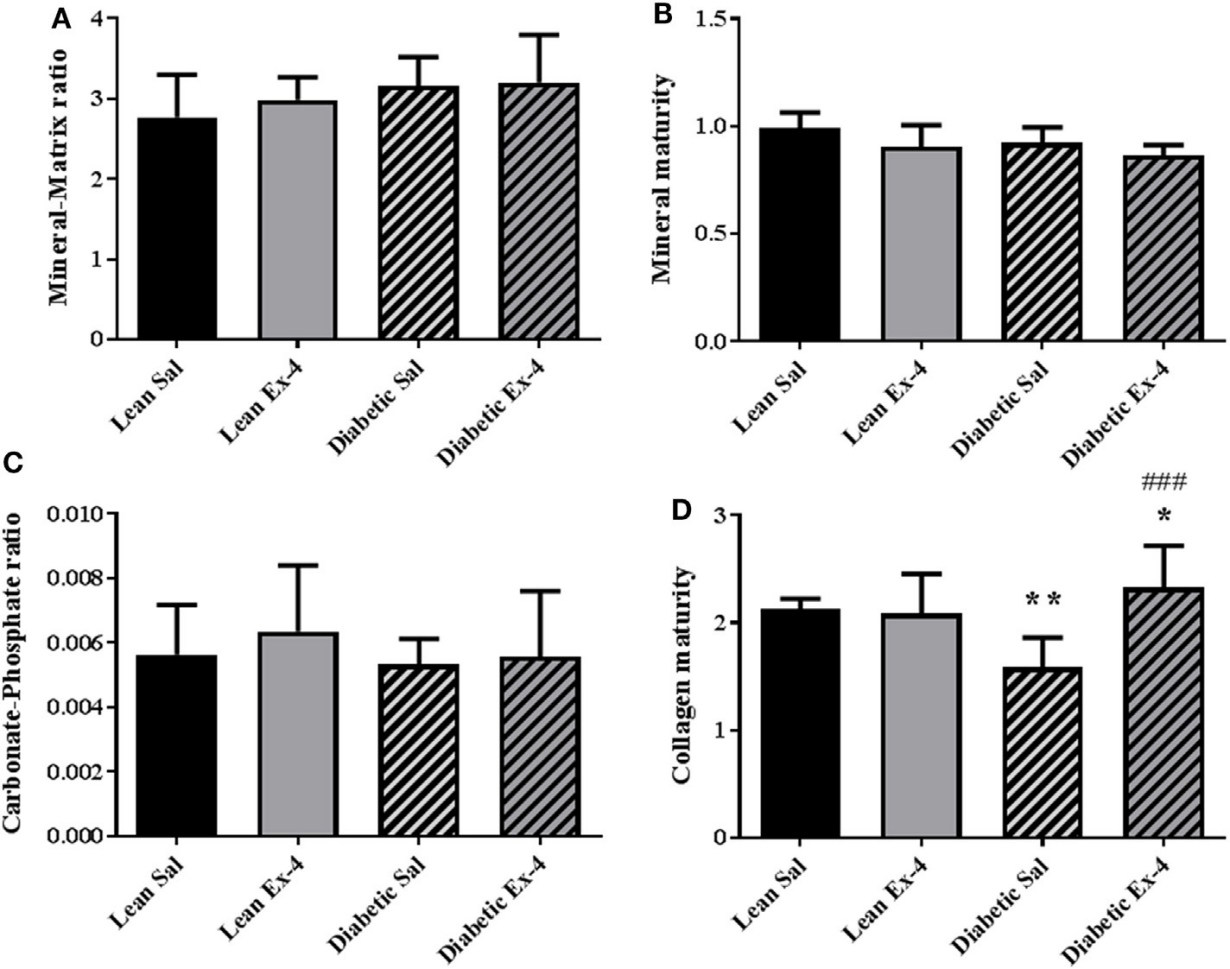
Effect of Exenatide on tissue material properties assessed at bone formation sites in db/db and control mice. Measurements were performed by Fourier-transform infrared microscopy between the fluorescent labels of the trabecular region of tibial sections in 13-week-old db/db and lean mice treated with either Exenatide or saline. **(A)** Mineral-to-matrix ratio. **(B)** Mineral maturity. **(C)** Carbonate-to-phosphate ratio. **(D)** Collagen maturity index. Bars represent mean ± SD of *n* = 10 mice/group, *P < 0.05, **P < 0.01 versus lean Sal; ^###^*P* < 0.001 versus diabetic Sal.

Because there was no alteration in the calcium content of cortical bone in db/db mice, tissue material properties were investigated at bone formation sites using FTIRM. Mineral/matrix ratio, mineral maturity, and carbonate/phosphate ratio were similar in the four groups of animals (Figures 7A–C). However, db/db mice showed lower collagen maturity (−25%, *P* < 0.01) than lean mice (Figure 7D). Treatment with Exenatide significantly increased collagen maturity (+47%, *P* < 0.001) in db/db mice but had no effect on lean mice (Figure 7D).

## Discussion

Among all complications of diabetes mellitus, bone frailty is often disregarded by diabetologists despite its high impact on life quality. Bone fragility is certainly a consequence of chronic hyperglycemia but can also result of direct or indirect effects of anti-diabetic drugs. With respect to the conflicting results on BMD in T2DM, the underlying mechanisms of bone fragility in T2DM seems to be related to the quality rather than the quantity of bone tissue, and as such, it is absolutely crucial to ascertain how marketed anti-diabetic drugs influence bone quality. In the present study, we thought to investigate how Exenatide, a GLP-1 receptor agonist, marketed for the treatment of type 2 diabetes, influences bone quality, remodeling, and strength in a murine model of genetically inherited T2DM that reproduces several features of human diabetes. Four weeks’ treatment with Exenatide was sufficient to improve trabecular bone in T2DM mice by increasing bone formation, which could possibly due to the observed acute increase in skeletal perfusion. While Exenatide did not show significant effect on bone strength and mineral, it improved the properties of the matrix by increasing the collagen maturity in site of new bone formation. *In vitro* data suggest that Exenatide can promote bone formation by osteoblasts when cultured only in high-glucose concentrations.

Leptin has been shown to induce a negative energy balance by reducing appetite and increasing energy expenditure (34). Although not all obese patients would develop diabetes, most patients with T2DM are obese (35). Leptin circulates in serum at levels that mirrors body fat. However, obese individuals have been found to be resistant to leptin action (36). The long form of the leptin receptor is encoded by the *db* gene and a recessive mutation in this gene has been shown to affect the intracellular domain of the single membrane-spanning receptor (37). Although leptin appears to bind to its receptor in mutant homozygous *db/db* mice (38), defective intracellular signal transduction attenuates leptin function, leading to a characteristic phenotype of severe obesity and diabetes, similar to what is observed in most human T2DM patients (39, 40), and replicated in the present study as demonstrated by low-bone mass and severe diabetes. As such, although leptin can also have effects on bone independently of diabetes (41, 42), the db/db mouse represents a good model of T2DM, and reproduces the bone phenotype observed in most T2DM. In accordance with the literature, chronic administration of Exenatide reduced non-fasting plasma glucose in db/db mice (43). It also caused a slight reduction in the body mass of these mice, likely due to the effect of Exenatide on the central nervous system, where it enhances satiety and diminishes food consumption (44).

Previously, improvement in trabecular bone mass have been reported in diabetic rodents (cholecystokinin receptor A-deficient OLETF rats and Goto-Kakizaki rats) or rodent models of ovariectomy-induced osteoporosis in response to GLP-1RAs (7, 9, 11, 14). However, although our study confirms this finding in the leptin receptor-deficient mice, it also further augments our knowledge about the effects of such therapy on bone quality that is compromised in animal models of T2DM but also in human diabetic individuals. While Exenatide showed an anabolic effect in diabetic mice, it did not affect bone mass and quality in lean mice, similarly to a previous study (8). Exenatide reversed the low-osteoblast activity observed in saline-treated mice (augmentations of BFR/BS and MAR, no decrease in MS/BS) suggesting that this molecule acts on diabetic bone as an anabolic agent. Our results suggest also an uncoupling effect, in diabetes, as observed by the lack of effect on osteoclast surface, although it is not a measure of osteoclast activity. This finding seems in opposition to our previous published data that showed increased osteoclast surfaces and numbers with Exenatide in a mouse model of ovariectomy-induced osteoporosis (14). This could be attributed to the animal models used. The db/db mouse model is not solely an obese-diabetic model and alterations in bone turnover could be attributed to changes in leptin signaling and other hormonal levels. Furthermore, region-specific differences in bone turnover in db/db mice have been shown, with both bone formation and bone resorption being either decreased or increased (45). Unfortunately, we were not able to analyze osteoclastogenesis and *in vitro* bone resorption by osteoclasts isolated from db/db mice to clarify this inconsistency.

Furthermore, at site of bone formation, Exenatide was capable of augmenting collagen maturity that is compromised in saline-treated diabetic mice. The anabolic action of Exenatide can also be observed in the higher mineralization heterogeneity observed by qBEI that first restore levels similar to non-diabetic animals and second reflect the new bone formation that is not yet fully mineralized. The latter can also be evaluated from the lower, but not significant, Ca_mean_ values.

Taken all together, our results support the idea that Exenatide ameliorates the bone phenotype, in diabetic mice, through an increase in bone formation and that the drug has no effect in non-diabetic conditions. It is, however, still not very clear whether the increase in bone formation is due to direct effects of Exenatide on osteoblasts or to systemic effects. It has been shown that the GLP-1R is expressed in marrow mesenchymal cells including adipocytes and osteoblasts (14, 46), suggesting that the effects of Exenatide on bone formation could be direct by binding to the GLP-1R expressed on osteoblasts. Therefore, we tested the direct effects of Exenatide on osteoblasts *in vitro* in low- and high-glucose concentrations. Exposure of primary osteoblasts to high-glucose concentrations inhibited *in vitro* bone nodule formation, consistent with previous *in vitro* studies (47, 48). Addition of Exenatide in confluent primary osteoblasts cultured in normal glucose conditions did not affect bone nodule formation, in accordance with our previous findings showing that Exenatide has no effect on bone formation in non-hyperglycemic conditions (14). Studies conducted to examine the direct effects of GLP-1 agonists on *in vitro* bone formation have led to inconsistent results. Exenatide and Liraglutide were shown to promote proliferation and differentiation of a pre-osteoblastic cell line (MC3T3-E1) by direct binding to the GLP-1R, suggesting that GLP-1RAs could promote osteoblast-mediated bone formation (49–51). However, another study has yielded opposite results using the same cell line (52). However, the MC3T3-E1 cell line does not form trabecular bone nodules, contrary to primary osteoblasts used in our study. We cannot exclude, however, that Exenatide may exert a direct anabolic effect on bone by increasing proliferation and differentiation of osteoblasts rather than affecting their bone forming activity. Interestingly, Exenatide directly reduced the deleterious effect of glucose on bone formation in a dose-dependent manner, while it had no effect under normal glucose conditions. This is in agreement with our *in vivo* observation showing that Exenatide exerts no effect in lean mice. The mechanisms for the possible protective effects of GLP-1RAs against the adverse action of glucose on osteoblasts are still unclear. Previous data indicate that culture of osteoblasts in high-glucose concentrations increases the expression levels of GLP-1R mRNA (53), suggesting that GLP-1R may directly link bone and glucose metabolism in osteoblasts. Therefore, the observed increase in bone formation caused by Exenatide in the presence of high-glucose concentrations might be due to upregulated GLP-1R expression, which could in turn magnify the effect direct effect of GLP-1RAs on osteoblasts. A recent study shows that GLP-1RAs facilitate glucose uptake in skeletal muscle cells by activating the AMP-activated protein kinase (54). Consequently, GLP-1RAs could improve glucose uptake by osteoblasts therefore reducing glucotoxicity. This hypothesis needs, however, to be confirmed by future overexpression or knockout of GLP-1R studies and by testing of insulin signaling and glucose metabolism in osteoblasts in response to Exenatide.

Alternatively, GLP-1RAs may increase bone mass by a systemic effect such as an increase in bone blood flow. Patients with T2DM demonstrate cardiovascular complications, including endothelial dysfunction and impaired vasodilatation (55). Therefore, it would be expected that the blood flow to bone is reduced with diabetes. The reference method to measure bone blood flow is the intravascular injection of labeled microspheres (56). However, a strong correlation was found between the standardized measure of bone blood flow and hindlimb perfusion measured using LDI (57), indicating that measuring whole hindlimb blood flow is a good surrogate method. Similar to what has been observed in humans (55), our diabetic mice demonstrated impaired blood flow, indicated by a decrease in hindlimb perfusion. This is in accordance with a previous study that showed lower bone and bone marrow perfusion in Zucker diabetic rats and suggested that impairment of the bone circulation may contribute to the osteopenia observed in these rats (58). Furthermore, we have shown that Exenatide acutely increased hindlimb perfusion. Similar vasodilator effects of Exenatide have been demonstrated in *ex vivo* studies with dilation of the rat femoral artery (59) and thoracic aorta (60). However, only one clinical study performed in overweight men has shown a beneficial effect of Exenatide on capillary perfusion using the laser Doppler technique (61). Consistent with our data, the effects of Exenatide were observed rapidly, suggesting direct actions of Exenatide on vascular perfusion.

The increased bone formation observed in db/db mice treated with Exenatide could be attributed in part to the increased skeletal perfusion. Blood supply plays a key role in bone formation by transporting oxygen, osteoprogenitor cells, and growth factors released from endothelial cells, which control the recruitment, proliferation, differentiation, and function of osteoblasts and osteoclasts (62). Because vasodilation and angiogenesis both contribute to bone blood supply, changes in blood flow could indirectly affect bone formation. Since decreased bone formation occurs concurrent with reduced hindlimb perfusion in db/db mice, this suggests that insufficient perfusion could restrain bone formation. This contention is supported by the fact that elderly women with osteoporosis have reduced femoral blood flow (63). Increased perfusion resulting from Exenatide treatment could ameliorate this defect.

Our experiments were performed in male db/db mice that exhibit more severe clinical symptoms and we cannot exclude potential gender differences regarding the skeletal effects of Exenatide in db/db mice. T2DM women treated with Exenatide presented more benefit in terms of cardiovascular risk factor and body weight (64) that can be attributed to interaction of Exenatide with estrogen signaling (65). As there is increased fracture risk among T2DM women compared with men, it would be of interest to verify our findings in female T2DM models in order to have sex-specific treatment guidelines.

In conclusion, administration of Exenatide to leptin receptor-deficient db/db mice improves bone mass, microarchitecture, and quality possibly by (1) reversing the impaired bone formation induced by glucose, (2) stimulating bone formation systemically *via* increased hindlimb perfusion, and (3) improving collagen content. This study provides additional evidence for the inclusion of GLP-1RAs in therapeutic strategies for diabetic patients with concurrent bone disease.

## Author Contributions

Study design: MP, CC, and MC. Study conduct: MP, SG, and GM. Data collection: MP, SG, and GM. Data analysis: MP, SG, and GM. Data interpretation: MP, GM, JR, and CC. Drafting manuscript: MP and CC. Revising manuscript content: SG, GM, JR, AF, and MC. Approving final version of manuscript: MP, CC, SG, GM, JR, AF, and MC take responsibility for the integrity of the data analysis.

## Conflict of Interest Statement

The authors declare that the research was conducted in the absence of any commercial or financial relationships that could be construed as a potential conflict of interest. AF was employed by the company Transpharmation but did not provide any funding.
